# Network effects of Stanford Neuromodulation Therapy (SNT) in treatment-resistant major depressive disorder: a randomized, controlled trial

**DOI:** 10.1038/s41398-023-02537-9

**Published:** 2023-07-03

**Authors:** Jean-Marie Batail, Xiaoqian Xiao, Azeezat Azeez, Claudia Tischler, Ian H. Kratter, James H. Bishop, Manish Saggar, Nolan R. Williams

**Affiliations:** 1 Stanford Brain Stimulation Lab, Stanford, CA USA; 2 Pôle Hospitalo-Universitaire de Psychiatrie Adulte, Centre Hospitalier Guillaume Régnier, Rennes, France; 3 Baylor College of Medicine, Houston, TX USA; 4 Department of Psychiatry and Behavioral Sciences, Stanford University, Stanford, CA USA

**Keywords:** Predictive markers, Depression

## Abstract

Here, we investigated the brain functional connectivity (FC) changes following a novel accelerated theta burst stimulation protocol known as Stanford Neuromodulation Therapy (SNT) which demonstrated significant antidepressant efficacy in treatment-resistant depression (TRD). In a sample of 24 patients (12 active and 12 sham), active stimulation was associated with significant pre- and post-treatment modulation of three FC pairs, involving the default mode network (DMN), amygdala, salience network (SN) and striatum. The most robust finding was the SNT effect on amygdala-DMN FC (group*time interaction *F*(1,22) = 14.89, *p* < 0.001). This FC change correlated with improvement in depressive symptoms (rho (Spearman) = −0.45, df = 22, *p* = 0.026). The post-treatment FC pattern showed a change in the direction of the healthy control group and was sustained at the one-month follow-up. These results are consistent with amygdala-DMN connectivity dysfunction as an underlying mechanism of TRD and bring us closer to the goal of developing imaging biomarkers for TMS treatment optimization.

**Trial registration**: ClinicalTrials.gov NCT03068715

## Introduction

Failure to achieve complete remission in treating Major Depressive Disorder (MDD) leads to increased morbidity, relapse rates [1], and suicidality [2]. Elucidating the neural mechanisms associated with treatment response could improve therapeutics—via an approach based on personalized psychiatry [3]—and better our understanding of the neurophysiology of treatment-resistant depression (TRD). Biological mechanisms in MDD have been investigated using network-level whole-brain approaches, such as functional connectivity (FC) imaging, which has led to a better understanding of biological mechanisms of MDD [3, 4]. Three core circuits have been identified in depression: the medial prefrontal–posterior cingulate default mode network (DMN), the fronto-parietal central executive network (CEN), and the cingulo-opercular salience network (SN) [5]. The latter two are known to have an inhibitory function over the DMN and a crucial role in the regulation of cognitive and emotional processes [5]. Recent findings have shown that in patients with depression, the DMN has a greater FC with dorsomedial prefrontal cortex (dmPFC) [6], subgenual ACC (sgACC) [7], and subgenual prefrontal cortex (sgPFC) than in healthy controls and is associated with rumination [8]. Some work has emphasized that cognitive control and reward-related networks are associated with treatment resistance in depression [9]. Both DMN FC and anterior insula glucose metabolism predict response to antidepressant (ATD) therapy, suggesting that there may be functional markers within both the DMN and the SN that can guide treatment selection such as pharmacotherapy or cognitive behavior therapy [10–12]. Interestingly, the literature on rTMS treatment response is converging toward using the anticorrelation between the DLPFC, a part of the CEN, and the sgACC to predict clinical outcomes [13–16]. Thus, some randomized controlled studies have emphasized that left or bilateral DLPFC TMS / intermittent Theta Burst Stimulation (iTBS) could be associated with modifications within and between FC of SN, CEN, and DMN. [17–20] Whereas one study failed to elicit such effect probably due to lack of statistical power [21]. However, whether TMS alters CEN and SN regulation of the DMN remains unclear.

Indeed, relatively little is known about brain functional correlates of ATD treatment. Most studies exploring this question utilize typical ATD therapies, which are inherently confounded by nonspecific factors due to the duration of time between initiation and ATD effect. More recently, other pharmacological agents like ketamine and psilocybin have demonstrated rapid ATD effects, suggesting that they may be able to help address this issue. However, the contribution of these drugs in the study of the mechanisms of action might be limited by non-specific pharmacological action [22, 23] and lack of effective blinding—i.e. poorly assessed and unsuccessful [24]—which impairs the generalizability of the findings. In the current study, we investigated resting state FC changes observed immediately and one-month after a course of an accelerated, high-dose, intermittent theta burst stimulation (iTBS) protocol with functional MRI-guided targeting—termed Stanford Neuromodulation Therapy (SNT) [25]. This protocol resulted in a high remission rate of 57.1% immediately after five days of treatment and 46.2% remission at one-month post-treatment [25]. While the efficacy of TMS in general [26] and SNT [25, 27] in particular have been investigated, less is known about the downstream neural effects of treatment response. Therefore, the efficacy, rapidity, and durability of SNT suggest that it is an ideal therapeutic paradigm to investigate functional changes associated with treatment response. Furthermore, the recent RCT with an intact blind allows us to investigate the downstream effects of ATD treatment while controlling for many of the concerns noted above. We aimed to address the following mechanistic questions: What are the acute effects of SNT therapy on resting state FC? What is the relationship between stimulation-induced FC changes and clinical changes? How does participant resting-state FC compare to that of healthy controls? Are these alterations sustained over one-month post-treatment?

To this end, we investigated the effects of active Stanford Neuromodulation Therapy—as compared to sham stimulation—on FC immediately after 5 days of treatment. Next, we examined the relationship between FC changes and clinical changes from baseline to the immediate-post visit. Finally, we explored whether baseline FC was different from that of a healthy control group, and if so, whether active stimulation could normalize it. We hypothesized that active stimulation would be associated with significant changes in FC from baseline to the immediate-post visit and that these FC changes would be associated with the extent of clinical change. As exploratory hypotheses, we postulated that 1/the FC at baseline would differ between TRD participants and healthy controls, and the FC changes induced by active stimulation would eliminate this difference 2/ the induced FC changes in the active group immediately post-stimulation would be sustained at one-month. In this paper, the overarching goal was to explore regional/distant functional brain response and its relationship with clinical changes.

## Methods

### Participants

Detailed information regarding the subjects, experimental design, and clinical data analysis have been previously reported [25]. Briefly, 32 subjects with a sole primary diagnosis of MDD were recruited for this double-blinded, sham-controlled RCT, and 29 participants who continued to meet inclusion criteria received either active (*N* = 14) or sham (*N* = 15) SNT. We enrolled participants suffering from a moderate to severe depressive episode (Hamilton Depression Rating Scale [HDRS] ≥ 20 and Montgomery and Åsberg Depression Rating Scale [MADRS] ≥20) and were required to have not responded to at least one antidepressant medication (minimum trial duration of six weeks) to be eligible for the study. All participants were TMS naïve. 5 of 29 subjects were excluded from the current analyses because of insufficient MRI quality (see MRI section for further details). The one-month MRI was not obtained for 2 subjects (i.e., only baseline and immediate-post follow-up scans were obtained). In summary, 24 subjects (12 active, 12 sham) were included in the analyses that include the immediate-post follow-up, and 22 subjects were included in the analyses that include the one-month follow-up (for CONSORT diagram see supplementary figure S1). For our exploratory analyses, we enrolled a healthy control (HC) group of 22 subjects (17 female), for descriptive statistics see results section. Healthy participants were provided with informed consent. The Mini International Neuropsychiatric Interview was performed on all healthy participants. Exclusion from the study was any history of psychiatric or neurological disorders (depression and chronic pain, substance abuse), left handness, pregnancy, and MR incompatibility and those affiliated with the university students, faculty, staff). Each HC was scanned once, and clinical assessment (see below) was administered at the time of scan to confirm the absence of psychiatric disorders. All participants provided informed consent for the study prior to participation, and all procedures were approved by the Stanford School of Medicine Institutional Review Board.

### Stanford Neuromodulation Therapy (SNT)

Participants received active or sham STN, a 5-day, high-dose, accelerated iTBS treatment protocol that has been described in detail previously [25]. The baseline resting state fMRI scan was used to determine the location of the personalized target within the left dorsolateral prefrontal cortex (lDLPFC) for stimulation according to an algorithm that selects the area within the lDLPFC with the most negative FC with the sgACC, again as described in previous publications [25, 27].

### Clinical assessment

Participants’ depressive symptoms were assessed using the MADRS [28]. MADRS assessments were performed at the baseline, immediate-post and one-month follow-up visits.

### MRI image data acquisition and preprocessing

All participants were screened for MRI safety prior to any scanning procedures. MRI scans were acquired, and clinical assessments were performed on the same day. Each participant underwent identical baseline and post-treatment MRI scans consisting of structural and resting-state functional MRI acquisitions. All MRI scans were acquired using a 3TGE Discovery MR750 scanner with a 32-channel head-neck imaging coil at the Center for Cognitive and Neurobiological Imaging at Stanford.

High-resolution structural images using GE’s BRAVO sequence (three-dimensional, T1-weighted) were acquired for the whole brain (FOV = 256 × 256mm; matrix=256 × 256 voxel; slice thickness = 0.9 mm; TR = 2530 ms, TE = 2.98 ms, flip angle = 7°). During the 8-min resting state scan, participants were instructed to keep their eyes open, and their attention focused on a central fixation point, which consisted of a black screen with a white fixation cross.

Participants were also instructed to let their minds wander freely and avoid repetitive thoughts. Whole-brain resting-state scans were collected with a 3X simultaneous multi-slice (i.e., multiband) acquisition echo planar imaging (EPI) sequence: TR = 2000ms, TE = 30 ms, flip angle = 77°, slice acceleration factor = 3, FOV=230 × 230mm, matrix = 128 × 128 voxel, 1.8 × 1.8 mm2 in-plane resolution, 87 contiguous axial slices parallel to the anterior commissure–posterior commissure line, yielding >1.4 M voxels every 2 s. The head motion of participants was effectively minimized using memory foam and inflatable padding. Participant alertness during the resting state task was monitored using in-scanner video cameras.

As noted above, 5 subjects were excluded (see CONSORT diagram—Fig. S1 supplementary material) from the analyses because of large head movement during the scanning (mean fd > 0.2 mm, or max fd > 5 mm or more than 40% of data points FD > 0.5 mm). Hence this report included 12 participants in the active arm and 12 participants in the sham arm for the immediate-post analyses, and 11 participants in each arm for the one-month analyses.

MRI data were preprocessed using FMRIPREP version 20.2.025 [RRID:SCR 016216]. Each T1-weighted (T1w) volume was corrected for intensity non-uniformity using N4BiasFieldCorrection v2.1.0 [29] and skull-stripped using antsBrainExtraction.sh v2.1.0 (using the OASIS template). Spatial normalization to the ICBM 152 Nonlinear Asymmetrical template version 2009c [[30], RRID:SCR 008796] was performed through nonlinear registration with the antsRegistration tool of ANTs v2.1.0 [[31], RRID:SCR 004757], using brain-extracted versions of both T1w volume and template. Brain tissue segmentation of cerebrospinal fluid (CSF), white-matter (WM) and gray-matter (GM) was performed on the brain-extracted T1w using fast [32] (FSL v5.0.9, RRID:SCR 002823).

Resting state data were motion-corrected using mcflirt (FSL v5.0.9 [33]). This was followed by co-registration to the corresponding T1w using boundary-based registration [34] with six degrees of freedom using flirt (FSL). Motion correcting transformations, BOLD-to-T1w transformation and T1w-to-template (MNI) warp were concatenated and applied in a single step using antsApplyTransforms (ANTs v2.1.0) using Lanczos interpolation. Framewise displacement [35] was calculated for each functional run using the implementation of Nipype. All volumes with framewise displacement (FD) greater than 0.5 mm were excluded. ICA-based Automatic Removal Of Motion Artifacts (AROMA) was used to generate aggressive noise regressors and create a variant of data that is non-aggressively denoised [36]. The average signal within anatomically-derived eroded CSF and WM masks were included as confounder regressors. All data were spatially smoothed (6-mm-full-width, half-maximal Gaussian kernel) and temporal bandpass filtered (0.01–0.1 Hz). Data were detrended using Nilearn [37].

### Regions of interest (ROI)

ROIs were pre-defined from brain cortical and subcortical regions. Cortical parcellation was extracted from Schaefer’s brain parcellation [38]. We used the 100 parcels version and included 7 networks: CEN, DMN, SN, dorsal attention network (DAN), limbic network (LN), somatomotor network (SMN), and visual network (VN). The subcortical regions we predefined for further analysis included: the amygdala (AMY), striatum (STR), thalamus (THAL) and hippocampus (HIP). The bilateral executive, sensorimotor and limbic sub-regions of the striatum were predefined using the Oxford-GSK-Imanova Striatal Connectivity Atlas (striatum-con-label-thr25-3sub) [39]. We extracted sub-regions of the amygdala (AMY) using the Juelich histological atlas. There were six ROIs in total: the bilateral centro-median amygdala (CMA), bilateral latero-basal amygdala (LBA) and bilateral superficial amygdala (SA) [40]. Bilateral thalamus and hippocampus were extracted from the Harvard-Oxford cortical and subcortical structural atlases (FSL v5.0.9 [33]). The threshold used for the Juelich and Harvard-Oxford ROIs was 25%. A total of 116 ROIs were used. The full ROIs list is available as table s9 in the supplementary materials.

### Statistical analysis

All statistical analyses were conducted with R v4.2.1 [41].

Functional Connectivity (FC) was calculated as the Pearson correlation coefficient of the brain activity time series pairwise for all 116 × 116 ROIs (seeds). All Pearson correlation coefficients were then transformed into Fisher’s Z scores for further analysis, which are reported as our main FC outcome.

To test our primary hypothesis, we first determined which FC pairs were significantly altered by active as compared to sham SNT stimulation from baseline to immediate-post. As a second step, we looked at the relationship between FC changes and clinical changes. Finally, we performed several exploratory analyses: (1) we assessed the sustainability of SNT-induced FC alterations at one-month following treatment, and (2) we compared our TRD group to a healthy control group to determine if there were baseline differences, and if so, whether those differences were affected by SNT treatment.

We conducted our statistical analyses as follows:

1. 2 (active and sham groups) × 2 (baseline and immediate-post visits) repeated measures ANOVA with statistical significance set at *p* ≤ 0.001. Then, on significant FC pairs, Post Hoc analyses were corrected for multiple comparisons across all comparisons (active—sham * baseline—immediate-post) using the Bonferroni correction (*p* ≤ 0.05).
2. Spearman correlation tests, on the significant group*time interaction FC pairs that survived to Bonferroni correction, between FC changes and absolute MADRS changes from baseline to the immediate-post visit were conducted.
3. Exploratory hypotheses testing on the significant group*time interaction FC pairs that survived to Bonferroni correction. ANOVAs—followed by post-hoc *t*-test Bonferroni correction—were conducted to compare mean FC z-scores between baseline, immediate-post, one-month visits and the healthy control group.

## Results

### Treatment outcomes

Demographic information of the study subjects is summarized in Table S1 (supplementary material). In this cohort there were no significant differences in clinical characteristics between the sham and active groups, except for baseline MADRS scores (higher in the sham group). Therefore, baseline MADRS scores, in addition to gender, age, Maudsley score, duration of MDD, were added to our regression models as covariate in hypothesis 1 testing (2 × 2 ANOVA). To make sure the clinical efficacy of SNT in the subset of participants included here, with neuroimaging data, we then tested the effects of group (active/sham), time (baseline/immediate-post/one-month), and group by time interaction on MADRS total score using repeated measures ANOVA. In line with our previous publication [25], we found a significant effect of group (*F*(1,20) = 28.76, *p* < 0.001), time (*F*(2,42) = 19.72, *p* < 0.001), and group*time interactions (*F*(2,42) = 6.92, *p* = 0.003). A description of the whole population can be found in our previous publication [25].

The HC group had a mean age of 41.7 years (range = 21–69 years), all of whom underwent the same resting state scan protocol as the MDD cohort. At baseline, the HC group did not differ from the active group with respect to age (HC = 41.77 ± 12.99; active group = 50.99 ± 15.17, *t*(32) = 1.86, *p* = 0.071). The HC group had more females as compared to the active group (77.27% vs 22.73%).

Finally, we tested the effectiveness of our blinding. At the end of treatment day 1, one-way *t* tests indicated no significant differences from chance (chance guess metric = 0.50) in the sham (mean guess metric = 0.34, *p* = 0.19) and active (mean guess metric = 0.53, *p* = 0.79) treatment groups. At treatment day 5, one-way *t* tests indicated no significant differences from chance (chance guess metric = 0.50) in the sham (mean guess metric = 0.39, *p* = 0.56) and active (mean guess metric = 0.42, *p* = 0.52) treatment groups. There was no relationship between the change in guess metric—between treatment day 1 and 5—and the change in MADRS scores—between baseline and immediate-post visit (*r* = −0.22, *p* = 0.44).

### FC changes from baseline to post-treatment

**Fig. 1:**
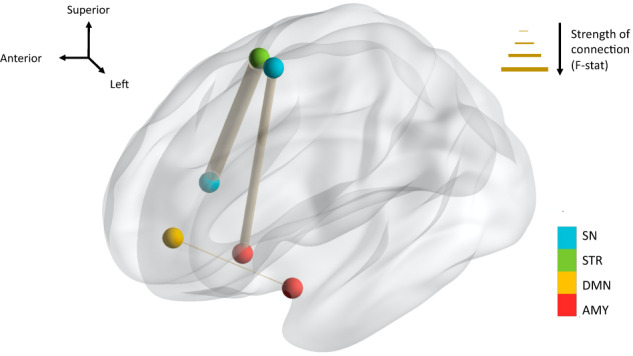
Glass brain visualization of the three FC pairs that survived Bonferroni correction (Table 1). Networks name: AMY: amygdala, DMN: default mode network, SN: salience network, STR: striatum. The thickness of each connecting edge is representative of the F-statistic, strength of connection, between corresponding seeds.

In this analysis, we tested which FC pairs were significantly altered by active as compared to sham SNT stimulation from baseline to immediate-post. 8 of 6670 FC pairs elicited a significant group (active vs sham) * time (baseline vs immediate-post) interaction (see supplementary Table S2). Three pairs survived to post-hoc Bonferroni correction (Table 1, Fig. 1). Two of them involved the amygdala ROI, with significant pre-post decrease FC right AMY (superficial) and left SN (medial 3) and increase FC between left AMY (superficial) and left DMN (prefrontal cortex 3). One of them involved the striatum network with significant pre-post increase FC between right STR (sensorimotor) and right SN (frontal-operculum-insula 1). Note that sham stimulation did not induce any significant pre-post change in FC for the left **AMY** (superficial)—left **DMN** (prefrontal cortex 3) pair (z-value = −2.04, *p* = 0.245) or the right **STR** (sensorimotor)—right **SN** (frontal-operculum-insula 1) pair (z-value = −1.55, *p* = 0.734) pair. Interestingly, sham stimulation was associated with a significant pre-post increase in FC of the right **AMY** (superficial)—left **SN** (medial 3) FC pair (z-value = 3.20, *p* = 0.008), whereas active stimulation induced a significant pre-post decrease in FC (z-value = −2.69, *p* = 0.04). Effect sizes (Cohen’s d) were calculated as a measure of the magnitude of the pre-post change (see supplementary Table S3).

**Table 1:** Summary of significant group (active vs sham) by time (baseline vs immediate-post) interaction—*p* ≤ 0.001 (not adjusted).

Seed 1	Seed 2	Pre-post effect in active group	F-value	*p*
LH **AMY** S^a^	LH **DMN** PFC3	➚	14.890	0.00085
RH **AMY** S^a^	LH **SN** Med3	➘	17.294	0.00041
RH **STR** sensorimotor^a^	RH **SN** FrOperIns1	➚	19.035	0.00025

*In bold* main network. *LH* left hemisphere, *RH* right hemisphere. Network Name: *AMY* amygdala, *DMN* default mode network, *SN* salience network, *STR* striatum. Nodes abbreviations: *PFC* prefrontal cortex, *Med* medial, *FrOperIns* frontal-operculum-insula, *S* superficial.

^a^These FC pairs survived post-hoc Bonferroni correction.

### Relationship between FC changes and depression severity changes from baseline to post-treatment

**Fig. 2:**
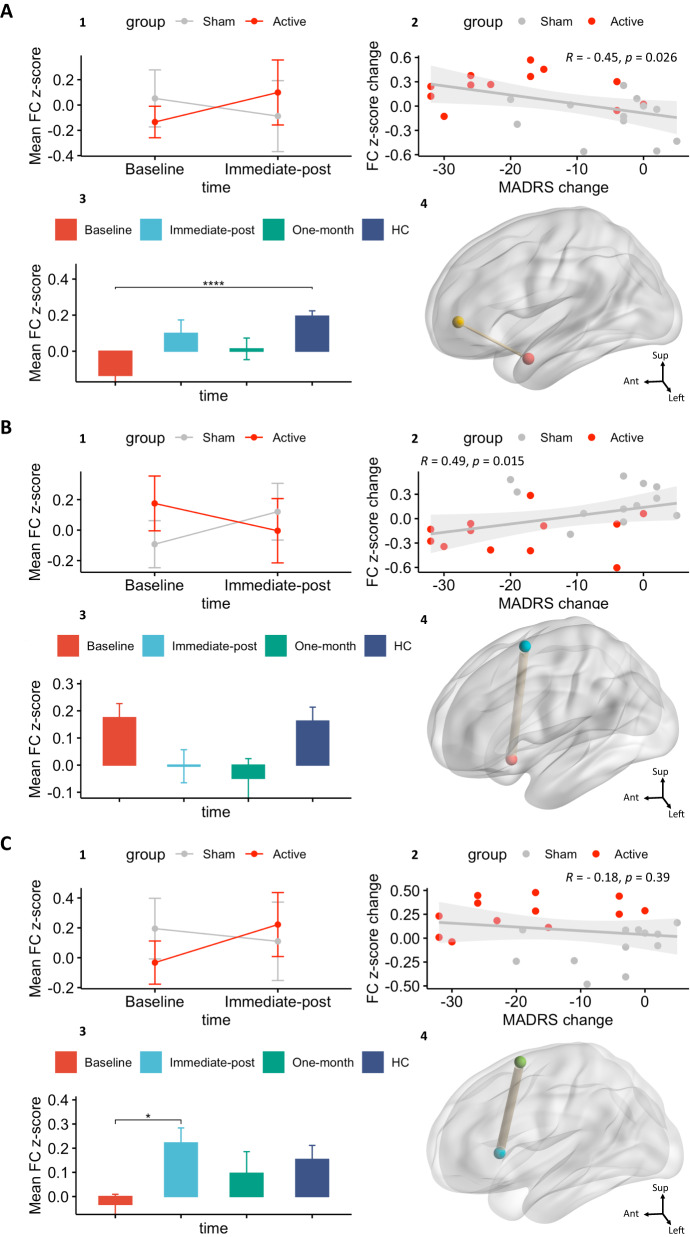
Grids summarizing the three FC pairs that demonstrated the strongest group*time interaction, relationship between FC and clinical changes or trend to normalization to HC FC level (Bonferroni corrected). **A** LH **AMY** S—LH **DMN** PFC3, **B** RH **AMY** S—LH **SN** Med3, **C** RH **STR** sensorimotor—RH **SN** FrOperIns1. Panels 1: group*time interaction plot from baseline to immediate-post; Panels 2: Correlation between MADRS changes and FC changes from baseline to immediate-post; Panels 3: barplot illustrating mean FC z-score post-hoc comparisons between group FC across three timepoints and healthy control group; Panels 4: glass brain visualization of the corresponding FC pair. LH: left hemisphere, RH: right hemisphere. Networks name: AMY (red nodes): amygdala, DMN (yellow nodes): default mode network, SN (light blue nodes): salience network, STR (green nodes): striatum. The thickness of each connecting edge is representative of the F-statistic, strength of connection, between corresponding seeds. FC: functional connectivity; HC: healthy control group. ****p ≤ 0.001, *p ≤ 0.05.

In this analysis, we tested whether any FC changes induced by SNT were correlated with clinical changes. We found that changes in two of three FC pairs (Table 1) were significantly associated with change in depression severity as measured by the MADRS (Fig. 2). Specifically, the left **AMY** (superficial)—left **DMN** (prefrontal cortex 3) FC pair exhibited a negative correlation (rho(Spearman) = −0.45, df = 22, *p* = 0.026) between FC changes and MADRS changes. That is, the greater the increase in FC, the less depressed the participant became after SNT. Conversely, the right **AMY** (superficial)—left **SN** (medial 3) FC pair exhibited a positive correlation (*rho (Spearman)*=0.49, df = 22, *p* = 0.015) between FC changes and MADRS changes, meaning that the greater the decrease, the less depressed the participant became after SNT. No FC pairs demonstrated a statistically significant correlation with sham SNT.

### Exploratory hypotheses

We performed further analyses to test (1) whether FC at baseline differed between TRD participants and healthy controls and, if so, whether the FC changes induced by active stimulation eliminated this difference, and (2) if the induced FC changes in the active group immediately post-stimulation were sustained at one month.

### FC z-score comparison between active group post-treatment and HC

Two pairs (of three) exhibited immediate-post mean FC z-scores in the active group that were *not* statistically different from the mean FC z-scores in the healthy control group (Fig. 2). These pairs were the left **AMY** (superficial)—left **DMN** (prefrontal cortex 3) (*t*(14.56) = −1.19, Bonferroni corrected *p* = 1) and the right **STR** (sensorimotor)—right **SN** (frontal-operculum-insula 1) (*t* (27.57) = 0.81, Bonferroni corrected *p* = 1). Interestingly, the baseline left **AMY** (superficial)—left **DMN** (prefrontal cortex 3) FC z-score was significantly lower than the HC group (*t*(24.87) = −7.07, Bonferroni corrected *p* ≤ 0.001), suggesting that active stimulation changed AMY-DMN FC in the direction of the HC group. For more details on the post-hoc paired t-tests see supplementary Tables S6, S7, S8.

### FC z-score comparison between active group post-treatment and one-month

The three pairs that exhibited a significant group*time interaction from baseline to immediate-post did not show a statistically significant difference between immediate-post and one-month, suggesting a maintenance of the effect following active stimulation (left **AMY** (superficial)—left **DMN** (prefrontal cortex 3): *t*(21.07) = 0.9, Bonferroni corrected *p* = 1; right **AMY** (superficial)—left **SN** (medial 3): *t*(21.33) = 0.47, Bonferroni corrected *p* = 1; right **STR** (sensorimotor)—right **SN** (frontal-operculum-insula 1) *t*(19.56) = 1.16, Bonferroni corrected *p* = 1). Again, for more details on the post-hoc paired t-tests please see supplementary Tables S6, S7, S8.

## Discussion

The present study examined brain functional connectivity changes in TRD patients participating in a clinical trial of SNT. By using an RCT design and performing analyses of the interaction between the treatment effect (before vs. after treatment) and the group effect (active vs. sham), we explored the functional response to treatment while controlling for placebo effects. Our pre-defined ROI approach identified three seed pairs with statistically significant changes with SNT. First, amygdala-DMN FC significantly increased from baseline to immediate-post after active SNT as compared to sham, and greater increases correlated with better clinical outcomes. Subsequent exploratory analysis was consistent with a possible normalization of pathological baseline FC. Second, amygdala-SN FC significantly decreased from baseline to immediate-post after active SNT as compared to sham. Again, greater decreases correlated with better outcomes. Third, striatum-SN FC significantly increased from baseline to immediate-post after active SNT as compared to sham. These results offer a unique insight into the putative mechanisms underlying the ATD effect of SNT—a high dose, fMRI-guided, accelerated intermittent TBS protocol—in TRD. Overall, our study confirmed that from a dimensional perspective the DLPFC targeted TMS is efficacious on depressive symptoms, as demonstrated recently in a transdiagnosis meta-analysis [42], and evidenced that the antidepressant effect associated with SNT protocol may be mediated by regional brain FC within DMN, amygdala, striatum, and SN.

Our findings highlight a central role of altered DMN FC as a mechanism of therapeutic effect and raise the possibility that this change could serve as a biomarker of therapeutic outcomes following SNT. DMN FC has been frequently reported to be abnormal in MDD but with inconsistencies in the differences observed [6, 43]. For example, contrary to the currently favored hypothesis of DMN hyperconnectivity in MDD [6], one recent large trial reported a decrease within DMN FC to be characteristic of patients suffering from recurrent MDD as compared to healthy controls [43]. Further, DMN functional activity has been linked with key MDD clinical components such as self-referential thinking [6, 44] and is hypothesized to be involved in pharmacological and nonpharmacological antidepressant mechanisms of action [45–48]. Increases within and between DMN intrinsic connectivity have been elicited in patients who remitted from depression [49], and an association between DMN-related connectivity and response—to both DLPFC [18, 50] and DMPFC [51] TMS—also has been reported. Notably, in Liston and al. study, baseline FC between sgACC and DMN was abnormally elevated in MDD patients, as compared to controls, and a full 5-week course of conventional TMS normalized this abnormal FC pattern [50]. Others have reported that the ATD effect following ketamine [52] or ATD medications [53] is associated with decreased DMN-CEN connectivity, while another study found an increased DMN-STR FC after 2 weeks of duloxetine treatment with FC changes correlating with symptom improvement [54]. Lastly, ketamine infusions (as compared to placebo) increased DMN-SN connectivity, which normalized as compared to healthy controls [55].

Antidepressant effects previously have been associated with a decrease within DMN FC [5, 10–12] as well as between DMN FC (CEN [53] and STR [54]), but we did not detect any such changes here. One possible explanation is the conservative p-value threshold we chose (*p* ≤ 0.001) to reduce the risk of Type I errors. Indeed, had we used a less conservative p-value threshold (*p* ≤ 0.005) then a statistically significant decrease within DMN connectivity would have been found (supplementary Tables S4, S5). Note that the effect size for the within DMN connectivity change was moderate (Cohen’s *d* = −0.557, supplementary Table S5), again consistent with the notion that a larger sample size might have provided the necessary power to detect this putative effect of SNT.

Moreover, we found a large effect of active stimulation on the AMY-DMN connectivity (ES = −0.872) suggesting a strong downstream effect of active SNT on this network. In addition, SNT altered other amygdala-centered connectivity patterns. Namely, following the SNT course, amygdala FC was increased with the DMN and decreased with the SN. Both FC patterns were associated with clinical changes with negative and positive correlations, respectively. Note that amygdala-DMN mean FC at the immediate-post visit was not statistically different from HC FC and was sustained at the one-month follow-up. Together with abnormal baseline FC (as compared to HC), our results suggest a modification of amygdala-DMN FC in the direction of HC. Across all results, amygdala-DMN FC change demonstrated the most robust evidence in terms of relevance to the mechanism of action of SNT. Several pieces of evidence in the literature support our findings. Indeed, a decreased amygdala-DMN FC has been related to an ongoing major depressive episode [56, 57]. Moreover, abnormal amygdala connectivity with the affective network—hyperconnectivity with hippocampi/parahippocampi and bilateral ventromedial OFC as well as hypoconnectivity with bilateral insula and left caudate—has been identified as a marker of emotional dysregulation in depressed adults [57]. In our study, baseline amygdala—DMN FC was abnormally low as compared to the HC group. Active SNT changed this connectivity pattern which was not statistically different from HC anymore and was associated with a better outcome. This result suggests a modification of FC—in the direction of HC—of emotion regulation network in SNT-responsive patients and is consistent with a top-down regulation model that previously has been proposed [6, 7, 10]. This result is also consistent with the hypothesis that patients suffering from depression experience a higher sensitivity to negative stimuli [6]. Of note, our comparisons with a HC were exploratory and therefore must be cautiously interpreted, as the HC group differed from the active SNT group with respect to gender.

The salience network is another important network identified as modulated by SNT in our study. We found increased connectivity between the SN and the striatum that reached HC group connectivity levels at the immediate-post visit as well as at one-month follow-up. In addition, decreased connectivity with the amygdala was noted and was associated with better clinical outcomes. The SN is thought to be a crucial contributor to MDD pathophysiology given its involvement in depressive cognitions [58]. The SN is involved in the detection of environmental changes [3] and, if needed, it coordinates additional processing and initiation of appropriate cognitive control [3]. Substantial evidence points to the SN as a network relevant to many different ATD therapies, including TMS [18, 51]. First, some studies have reported that baseline low within SN FC might be predictive of low response to ATD [59, 60]. Second, insula baseline task-based hyperactivity has been reported to be predictive of ATD/chronotherapy response [60]. Third, increased SN FC is a marker of response to DLPFC TMS [61], which we also found to be the case for SNT. Fourth, DMPFC-TMS responders exhibited higher baseline SN-sgACC FC [62], which suggests that the SN FC change is associated with antidepressant response to cortical stimulation, regardless of the target. Fifth, recent work reported that only salience network segregation was associated with symptom improvement following active 10 Hz TMS, using a segregation measure defined as the relative strength of within-network connectivity compared to between-network connectivity [63]. This latter result highlights the importance of this network in the prediction of response to TMS. From a mechanistic standpoint, SN FC with other networks might mediate downstream effects of DLPFC-targeted TMS. Indeed, the relationship between TMS and resting connectivity may only be observed when very specific cortical systems, such as the SN, are activated [64]. Furthermore, there is evidence that DLPFC stimulation induces dopamine release in SN-corticostriatocortical (CSC) loop circuits [58]. The modulation of DLPFC—as an SN cortical—is thought to have downstream effect on SN-CSC loop and would be associated with ATD response [58]. Our study demonstrates that active SNT modulates the SN-CSC loop which supports the literature on the ATD mechanism of action of DLPFC TMS. Our findings highlight the central role of the SN for the ATD effect of FC-guided lDLPFC aiTBS. Taken together, the SN is likely to be a key hub of the propagation of the effects of TMS in general and SNT more specifically.

Our results must be interpreted considering several limitations. First, the small sample size might have affected our ability to detect additional relevant functional brain responses following SNT. Thus, we should mention that the amygdala-SN model was not well distributed residuals. A follow up study with a larger sample size is needed to replicate these findings and further explore the mechanistic basis of ATD response to FC-guided lDLPFC aiTBS. Second, our HC group was not matched by sex with our depressed patients, which may impact the results of those exploratory analyses in an unknown manner. Finally, we would like to highlight that in our depressed sample females represented 33.33% and 41.67% of both sham and active groups—respectively—which is an unusual sex ratio for this illness. Our results should be interpreted considering this characteristic of our sample.

To conclude, our recently published clinical trial demonstrated the high efficacy of SNT, an accelerated, high-dose, iTBS protocol with functional MRI-guided targeting, for TRD [25]. In this paper, we report the SNT-associated changes in the whole-brain functional organization. This study presented the unique opportunity to investigate the relationship between rapid ATD effects and alterations in FC. Specifically, we investigated the effect of active SNT—as compared to sham stimulation—on FC immediately after a course of 5 days of treatment. Then, we explored the relationship between FC changes and clinical changes from baseline to immediate-post visit as well as the maintenance of the effect at the one-month follow-up. We found that the FC changes induced by SNT are distributed across key networks involved in depression pathophysiology. While the modest sample size limited statistical power, some networks still exhibited strong group*time interactions, suggesting a specific alteration of brain FC following an active stimulation course. Our results can be summarized by three main findings: (1) FC changes support improved regulation of emotion and reward processing after SNT; (2) amygdala-centered network changes are central to the therapeutic effect of SNT; and (3) while not a primary objective of the study, we tested the promise of a relationship between functional connectivity and clinical-outcome and explored any FC change following active stimulation in comparison to a HC group. In conclusion, we report the first evidence of downstream effects on brain functional connectivity of a rapid acting and highly effective high dose, accelerated iTBS protocol. Further study with a greater sample size is warranted to confirm these preliminary results and to better understand the mechanism of action of SNT.

## Supplementary information

Supplemental material
