# Supplementary material for: Network effects of Stanford Neuromodulation Therapy (SNT) in treatment-resistant major depressive disorder: a randomized, controlled trial
Source: Transl Psychiatry. 2023 Jul 3;13:240. doi: 10.1038/s41398-023-02537-9 (PMC10318050; doi:10.1038/s41398-023-02537-9)
Supplement: Supplementary file 1 — Supplemental material [file 41398_2023_2537_MOESM1_ESM.docx]

# Supplementary Materials


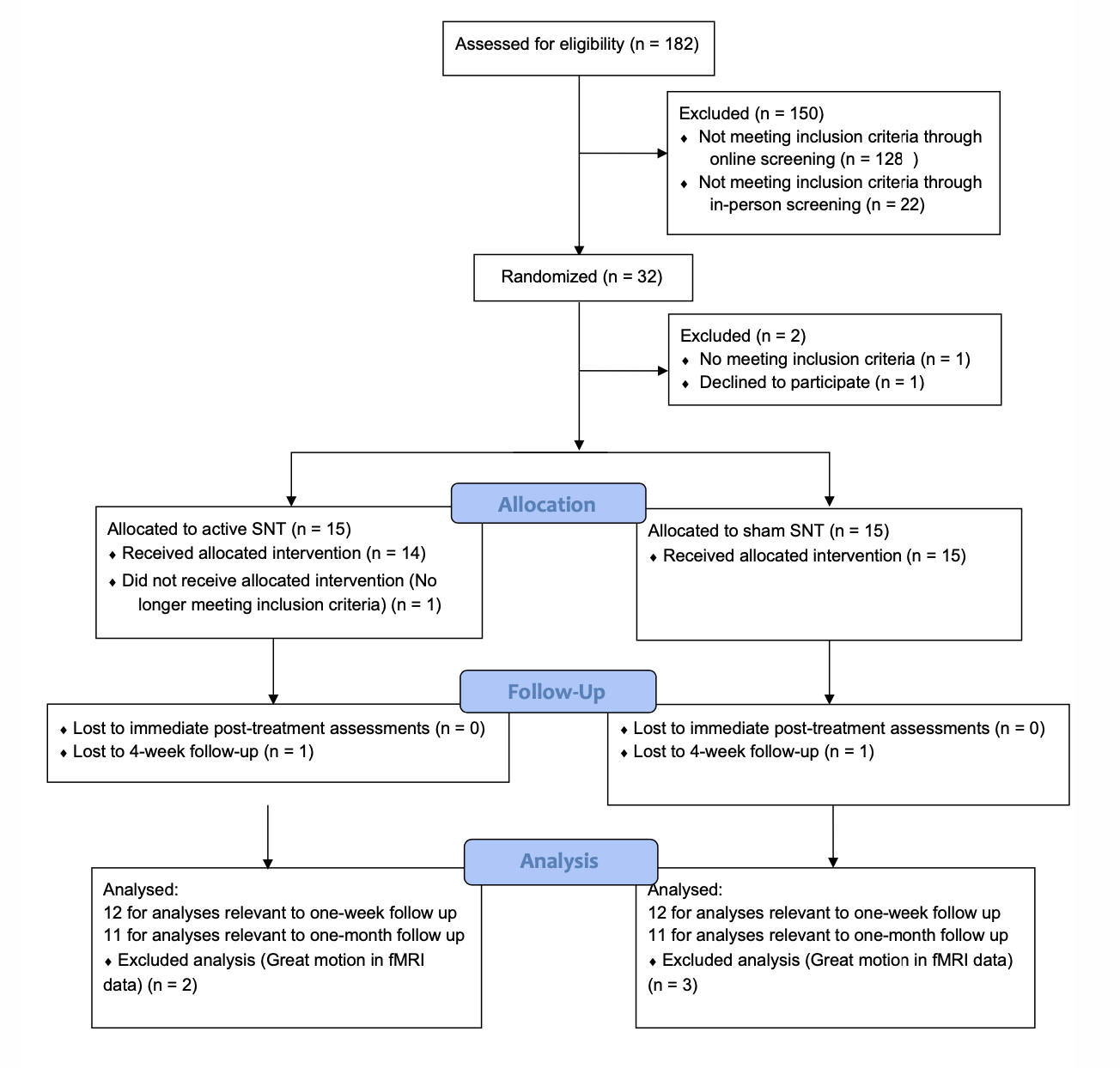


Figure s1: CONSORT diagram

| **Variable** | **N** | **Sham, N = 12^1^** | **Active, N = 12^1^** | **p-value^2^** |
| --- | --- | --- | --- | --- |
| **MADRS Baseline** | 24 |  |  | 0.028 |
| Mean (SD) |  | 36 (6) | 31 (4) |  |
| Range |  | 28 - 50 | 25 - 38 |  |
| **MADRS immediate-post visit** | 24 |  |  | <0.001 |
| Mean (SD) |  | 31 (9) | 12 (10) |  |
| Range |  | 15 - 47 | 2 - 28 |  |
| **MADRS one-month post visit** | 22 |  |  | 0.003 |
| Mean (SD) |  | 31 (10) | 13 (10) |  |
| Range |  | 12-48 | 4-33 |  |
| Unknown | 2 | 1 | 1 |  |
| **Age** | 24 |  |  | 0.9 |
| Mean (SD) |  | 50 (17) | 51 (15) |  |
| Range |  | 30 - 72 | 27 - 73 |  |
| **Gender** | 24 |  |  | >0.9 |
| Male |  | 8 / 12 (66.67%) | 7 / 12 (58.33%) |  |
| Female |  | 4 / 12 (33.33%) | 5 / 12 (41.67%) |  |
| **Maudsley Staging Model** | 24 |  |  | 0.682 |
| Mean (SD) |  | 9.08 (2.15) | 8.75 (1.77) |  |
| Range |  | 5-13 | 6-12 |  |
| **Duration of disease - lifetime (years)** | 24 |  |  | 0.2 |
| Mean (SD) |  | 22 (14) | 32 (18) |  |
| Range |  | 1 - 46 | 10 - 62 |  |
| ^1^ n / N (%) | | | | |
| ^2^ Wilcoxon rank sum test; Wilcoxon rank sum exact test; Fisher's exact test | | | | |

Table s1: Descriptive analysis of the sample

| **Seed 1** | **Seed 2** | **F-value** | **p-value** |
| --- | --- | --- | --- |
| LH **VN** 9 | LH **VN** 5 | 14.615 | 0.00093 |
| LH **DMN** PFC3 | LH **VN** 5 | 15.396 | 0.00073 |
| RH **VN** 6 | LH **DMN** PFC2 | 15.299 | 0.00075 |
| RH **DAN** Post4 | RH **SMN** 4 | 14.904 | 0.00085 |
| RH **DMN** PFCdPFCm1 | LH **VN** 5 | 17.462 | 0.00039 |
| LH **AMY** S | LH **DMN** PFC3 | 14.890 | 0.00085 |
| RH **AMY** S | LH **SN** Med3 | 17.294 | 0.00041 |
| RH **STR** sensorimotor | RH **SN** FrOperIns1 | 19.035 | 0.00025 |

Table s2: Summary of significant group (active vs sham) * time (baseline vs immediate-post) interaction - *p* ≤ 0.001. In bold, main network. LH: left hemisphere, RH: right hemisphere, AMY: amygdala, DAN: dorsal attention network, DMN: default mode network, SMN: somatosensory network, SN: salience network, STR: striatum, VN: visual network

| **Seed 1** | **Seed 2** | **Estimate** | **SE** | **z-value** | **p-value** | **ES Active pre-post** | **ES Active/Sham post** |
| --- | --- | --- | --- | --- | --- | --- | --- |
| LH **AMY** S | LH **DMN** PFC3 | 0.233 | 0.068 | 3.411 | 0.004 | 0.019 | -0.872 |
| RH **AMY** S | LH **SN** Med3 | -0.179 | 0.067 | -2.686 | 0.043 | 0.533 | 1.363 |
| RH **STR** sensorimotor | RH **SN** FrOperIns1 | 0.254 | 0.055 | 4.625 | 0.000 | -0.179 | -0.659 |

Table s3: Summary of significant FC pairs exhibited post hoc pre-post active analyses (Bonferroni corrected). In bold, main network. LH: left hemisphere, RH: right hemisphere, AMY: amygdala, DAN: dorsal attention network, DMN: default mode network, SMN: somatosensory network, SN: salience network, STR: striatum, VN: visual network. SE: residual standard deviation. ES: effect size.

Effect sizes (Cohen’s d) were computed to address the magnitude of pre-post (from baseline to immediate-post) active stimulation on FC pairs with ES range with small [0.019] to medium [0.533] effect and the magnitude of the active/sham with large effect [-0.659 - (-)1.942].

| **Seed 1** | **Seed 2** | **F** | **p** |
| --- | --- | --- | --- |
| LH VN 8 | LH VN 5 | 14.176 | 0.0010677 |
| LH VN 9 | LH VN 5 | 14.615 | 0.0009280 |
| LH SN FrOperIns1 | LH VN 5 | 10.559 | 0.0036762 |
| LH DMN Temp1 | LH VN 5 | 11.277 | 0.0028417 |
| LH DMN Temp2 | LH VN 7 | 12.412 | 0.0019149 |
| LH DMN PFC1 | LH SMN 1 | 10.079 | 0.0043854 |
| LH DMN PFC1 | LH CEN pCun1 | 12.834 | 0.0016604 |
| LH DMN PFC3 | LH VN 5 | 15.396 | 0.0007262 |
| LH DMN PFC5 | LH DAN FEF1 | 11.535 | 0.0025943 |
| LH DMN PFC6 | LH DMN Par2 | 10.127 | 0.0043075 |
| RH VN 2 | LH VN 5 | 12.999 | 0.0015708 |
| RH VN 4 | LH SN FrOperIns2 | 9.742 | 0.0049719 |
| RH VN 5 | LH VN 5 | 10.138 | 0.0042910 |
| RH VN 6 | LH VN 5 | 9.792 | 0.0048803 |
| RH VN 6 | LH DMN PFC2 | 15.299 | 0.0007485 |
| RH VN 7 | LH VN 5 | 12.805 | 0.0016762 |
| RH VN 8 | LH VN 5 | 14.336 | 0.0010142 |
| RH VN 8 | LH LN TempPole2 | 11.205 | 0.0029148 |
| RH VN 8 | LH DMN PFC2 | 11.560 | 0.0025717 |
| RH SMN 1 | LH DMN PFC6 | 11.502 | 0.0026242 |
| RH SMN 2 | LH DMN PFC3 | 12.361 | 0.0019489 |
| RH DAN Post1 | LH DAN FEF1 | 12.768 | 0.0016975 |
| RH DAN Post1 | LH CEN PFCl1 | 12.832 | 0.0016614 |
| RH DAN Post1 | RH VN 4 | 11.067 | 0.0030619 |
| RH DAN Post1 | RH VN 8 | 12.747 | 0.0017095 |
| RH DAN Post4 | RH SMN 4 | 14.904 | 0.0008468 |
| RH SN TempOccPar2 | RH DAN Post5 | 13.129 | 0.0015043 |
| RH SN FrOperIns1 | RH DAN Post1 | 10.600 | 0.0036224 |
| RH SN FrOperIns1 | RH DAN Post5 | 11.523 | 0.0026053 |
| RH SN Med1 | LH SN FrOperIns1 | 10.881 | 0.0032732 |
| RH CEN pCun1 | LH DAN FEF1 | 10.120 | 0.0043191 |
| RH DMN Temp1 | LH VN 5 | 11.501 | 0.0026253 |
| RH DMN Temp3 | LH DMN Par2 | 9.861 | 0.0047547 |
| RH DMN Temp3 | RH DAN Post2 | 10.691 | 0.0035053 |
| RH DMN PFCv1 | LH SMN 2 | 10.186 | 0.0042156 |
| RH DMN PFCdPFCm1 | LH VN 5 | 17.462 | 0.0003899 |
| LH dlPFC 1 | LH SMN 1 | 11.910 | 0.0022755 |
| LH dlPFC 1 | LH DAN FEF1 | 11.258 | 0.0028601 |
| LH dlPFC 1 | LH DMN PFC7 | 10.729 | 0.0034575 |
| LH dlPFC 1 | RH SMN 1 | 11.040 | 0.0030908 |
| LH AMY S | LH SN Med3 | 10.107 | 0.0043404 |
| LH AMY S | LH DMN Par1 | 10.263 | 0.0040978 |
| LH AMY S | LH DMN PFC3 | 14.890 | 0.0008507 |
| RH AMY S | LH SN Med3 | 17.294 | 0.0004096 |
| LH AMY CM | LH DAN PrCv1 | 10.766 | 0.0034115 |
| RH AMY CM | RH SN Med2 | 10.635 | 0.0035769 |
| RH AMY LB | LH SN Med3 | 9.751 | 0.0049552 |
| RH STR lim | LH DAN Post4 | 10.260 | 0.0041023 |
| RH STR lim | RH VN 8 | 9.745 | 0.0049659 |
| RH STR lim | RH DMN pCunPCC2 | 11.043 | 0.0030881 |
| LH STR exe | RH DMN pCunPCC1 | 11.759 | 0.0023986 |
| RH STR sensorimotor | RH SN FrOperIns1 | 19.035 | 0.0002486 |
| RH STR sensorimotor | RH STR | 10.791 | 0.0033810 |
| LH HIP 1 | LH VN 1 | 10.299 | 0.0040435 |
| RH THAL 1 | LH THAL 1 | 13.115 | 0.0015111 |

Table s4. Summary of significant group (active vs sham) * time (baseline vs immediate-post) interaction - p ≤ 0.005.

| **FC pairs** | **estimate** | **SE** | **z.value** | **p.value** | **ES Active pre-post** | **ES Active/Sham post** |
| --- | --- | --- | --- | --- | --- | --- |
| LH DMN Temp2.LH VN 7 | 0.241 | 0.076 | 3.161 | 0.009 | -0.009 | -0.777 |
| LH DMN PFC6.LH DMN Par2 | -0.357 | 0.109 | -3.286 | 0.006 | -0.557 | 0.518 |
| RH VN 7.LH VN 5 | -0.368 | 0.123 | -3.000 | 0.016 | -0.326 | 0.879 |
| RH SMN 2.LH DMN PFC3 | 0.211 | 0.060 | 3.488 | 0.003 | -0.384 | -0.633 |
| RH DAN Post1.LH CEN PFCl1 | 0.237 | 0.074 | 3.184 | 0.009 | 0.159 | -0.803 |
| RH SN TempOccPar2.RH DAN Post5 | -0.363 | 0.101 | -3.580 | 0.002 | -0.471 | 0.659 |
| RH SN FrOperIns1.RH DAN Post1 | 0.305 | 0.090 | 3.405 | 0.004 | 0.598 | -0.511 |
| RH SN FrOperIns1.RH DAN Post5 | -0.303 | 0.086 | -3.511 | 0.003 | -0.264 | 0.550 |
| RH SN Med1.LH SN FrOperIns1 | 0.296 | 0.087 | 3.401 | 0.004 | 0.248 | -0.539 |
| LH AMY S.LH SN Med3 | -0.244 | 0.088 | -2.780 | 0.033 | 0.282 | 0.895 |
| LH AMY S.LH DMN Par1 | 0.240 | 0.077 | 3.116 | 0.011 | 0.197 | -0.603 |
| LH AMY S.LH DMN PFC3 | 0.233 | 0.068 | 3.411 | 0.004 | 0.019 | -0.872 |
| RH AMY S.LH SN Med3 | -0.179 | 0.067 | -2.686 | 0.043 | 0.533 | 1.363 |
| RH STR lim.RH DMN pCunPCC2 | 0.230 | 0.068 | 3.392 | 0.004 | 0.188 | -0.558 |
| RH STR sensorimotor.RH SN FrOperIns1 | 0.254 | 0.055 | 4.625 | 0.000 | -0.179 | -0.659 |
| RH STR sensorimotor.RH STR | -0.180 | 0.059 | -3.028 | 0.015 | -0.577 | 0.690 |
| LH HIP 1.LH VN 1 | -0.192 | 0.069 | -2.760 | 0.035 | -0.576 | 0.758 |

Table s5. Summary of significant FC pairs exhibited post hoc pre-post active analyses (Bonferroni corrected) - initial ANOVA group*time interaction *p* threshold≤0.005. In bold, main network.

| **group1** | **group2** | **n1** | **n2** | **t-value** | **df** | **p** | **p.adj** |
| --- | --- | --- | --- | --- | --- | --- | --- |
| Baseline | Immediate-post | 12 | 12 | -2.83 | 15.90 | 1.20e-02 | 7.20e-02 |
| Baseline | One-month | 12 | 12 | -2.11 | 18.02 | 4.90e-02 | 2.94e-01 |
| Baseline | HC | 12 | 22 | -7.07 | 24.82 | 2.15e-07 | 1.29e-06**** |
| Immediate-post | One-month | 12 | 12 | 0.90 | 21.07 | 3.76e-01 | 1.00e+00 |
| Immediate-post | HC | 12 | 22 | -1.19 | 14.56 | 2.52e-01 | 1.00e+00 |
| One-month | HC | 12 | 22 | -2.72 | 16.47 | 1.50e-02 | 9.00e-02 |

Table s6. LH_AMY_S.LH_DMN_PFC3. Summary of mean FC z-score between left AMY (superficial) and left DMN (prefrontal cortex 3) post-hoc pair t-tests between baseline, immediate-post, one-month time points, and health control group (HC) with Bonferroni correction. ‘****’ *p* ≤ 0.001

| **group1** | **group2** | **n1** | **n2** | **t-value** | **df** | **p** | **p.adj** |
| --- | --- | --- | --- | --- | --- | --- | --- |
| Baseline | Immediate-post | 12 | 12 | 2.24 | 21.46 | 0.036 | 0.216 |
| Baseline | One-month | 12 | 12 | 2.51 | 19.88 | 0.021 | 0.126 |
| Baseline | HC | 12 | 22 | 0.17 | 28.65 | 0.864 | 1.000 |
| Immediate-post | One-month | 12 | 12 | 0.47 | 21.33 | 0.642 | 1.000 |
| Immediate-post | HC | 12 | 22 | -2.09 | 25.42 | 0.046 | 0.276 |
| One-month | HC | 12 | 22 | -2.37 | 21.80 | 0.027 | 0.162 |

Table s7. RH_AMY_S.LH_SN_Med3. Summary of mean FC z-score between right AMY (superficial) and left SN (medial 3) post-hoc pair t-tests between baseline, immediate-post, one-month time points, and health control group (HC) with Bonferroni correction.

| **group1** | **group2** | **n1** | **n2** | **t-value** | **df** | **p** | **p.adj** |
| --- | --- | --- | --- | --- | --- | --- | --- |
| Baseline | Immediate-post | 12 | 12 | -3.41 | 19.30 | 0.003 | 0.018* |
| Baseline | One-month | 12 | 12 | -1.30 | 15.57 | 0.211 | 1.000 |
| Baseline | HC | 12 | 22 | -2.62 | 32.00 | 0.014 | 0.084 |
| Immediate-post | One-month | 12 | 12 | 1.16 | 19.56 | 0.262 | 1.000 |
| Immediate-post | HC | 12 | 22 | 0.81 | 27.57 | 0.427 | 1.000 |
| One-month | HC | 12 | 22 | -0.54 | 20.23 | 0.595 | 1.000 |

Table s8. RH_STR_sensorimotor.RH_SN_FrOperIns1. Summary of mean FC z-score between right STR (sensorimotor) and right SN (frontal-operculum-insula 1) post-hoc pair t-tests between baseline, immediate-post, one-month time points, and health control group (HC) with Bonferroni correction.

‘*’ *p* ≤0.05


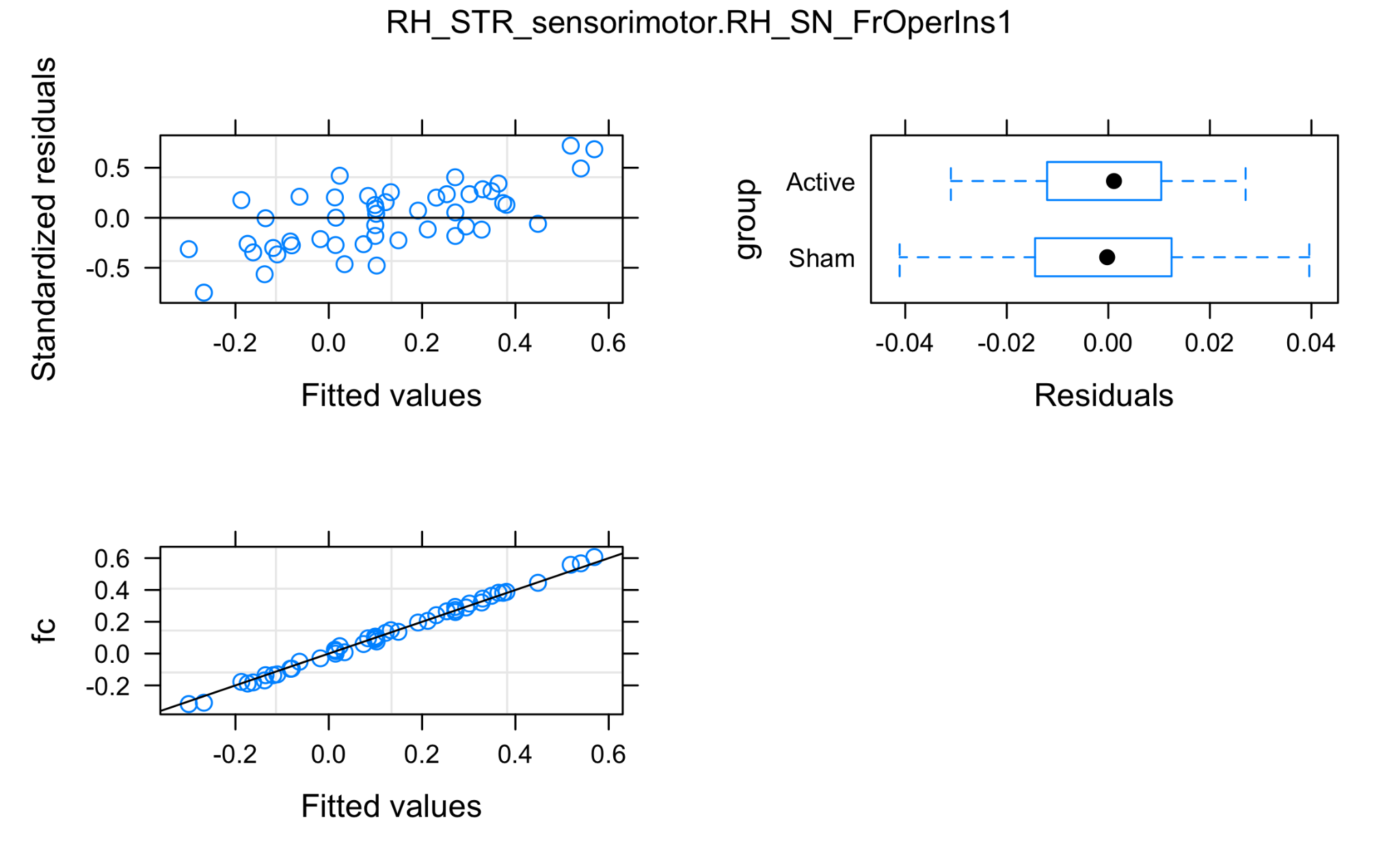


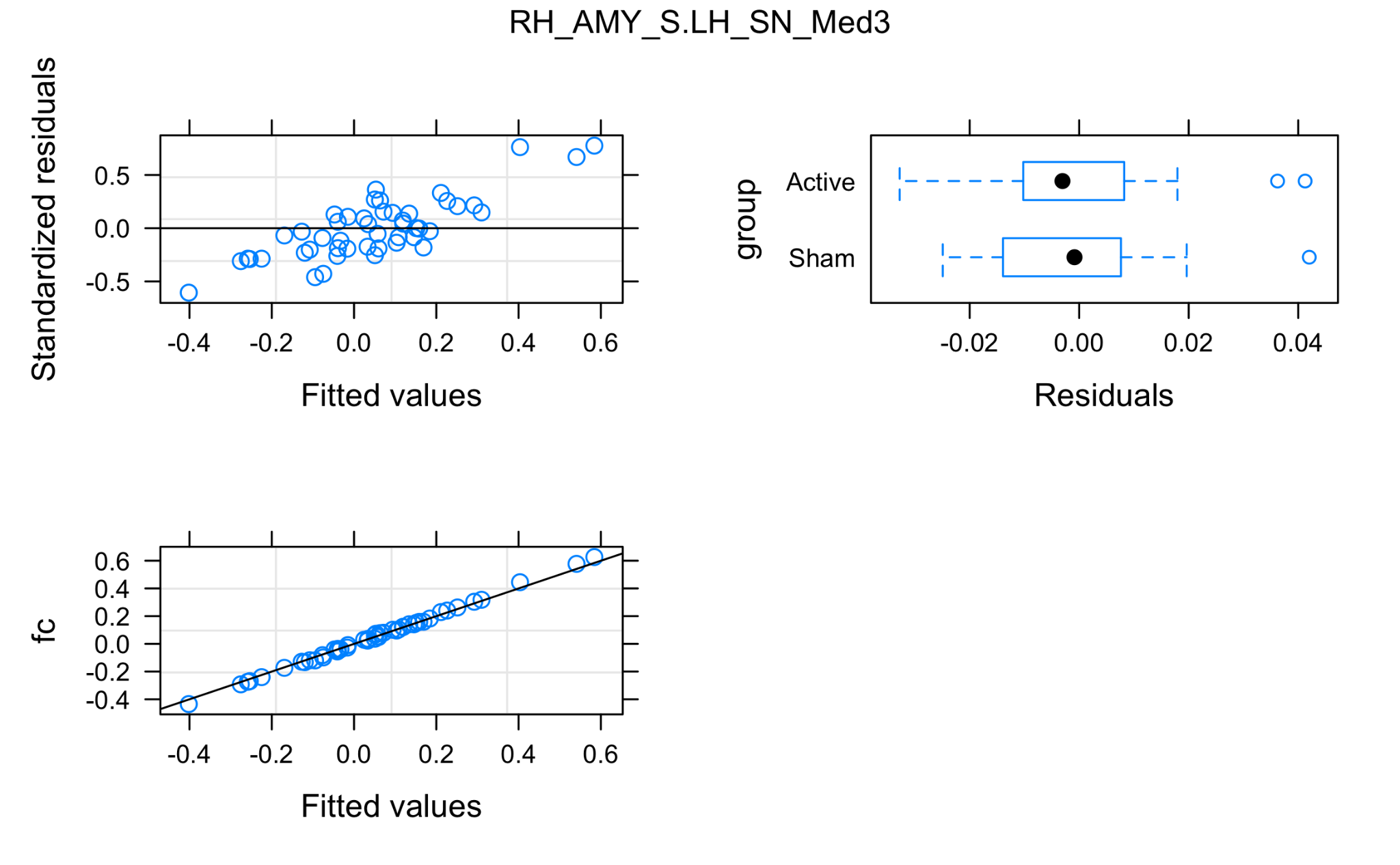


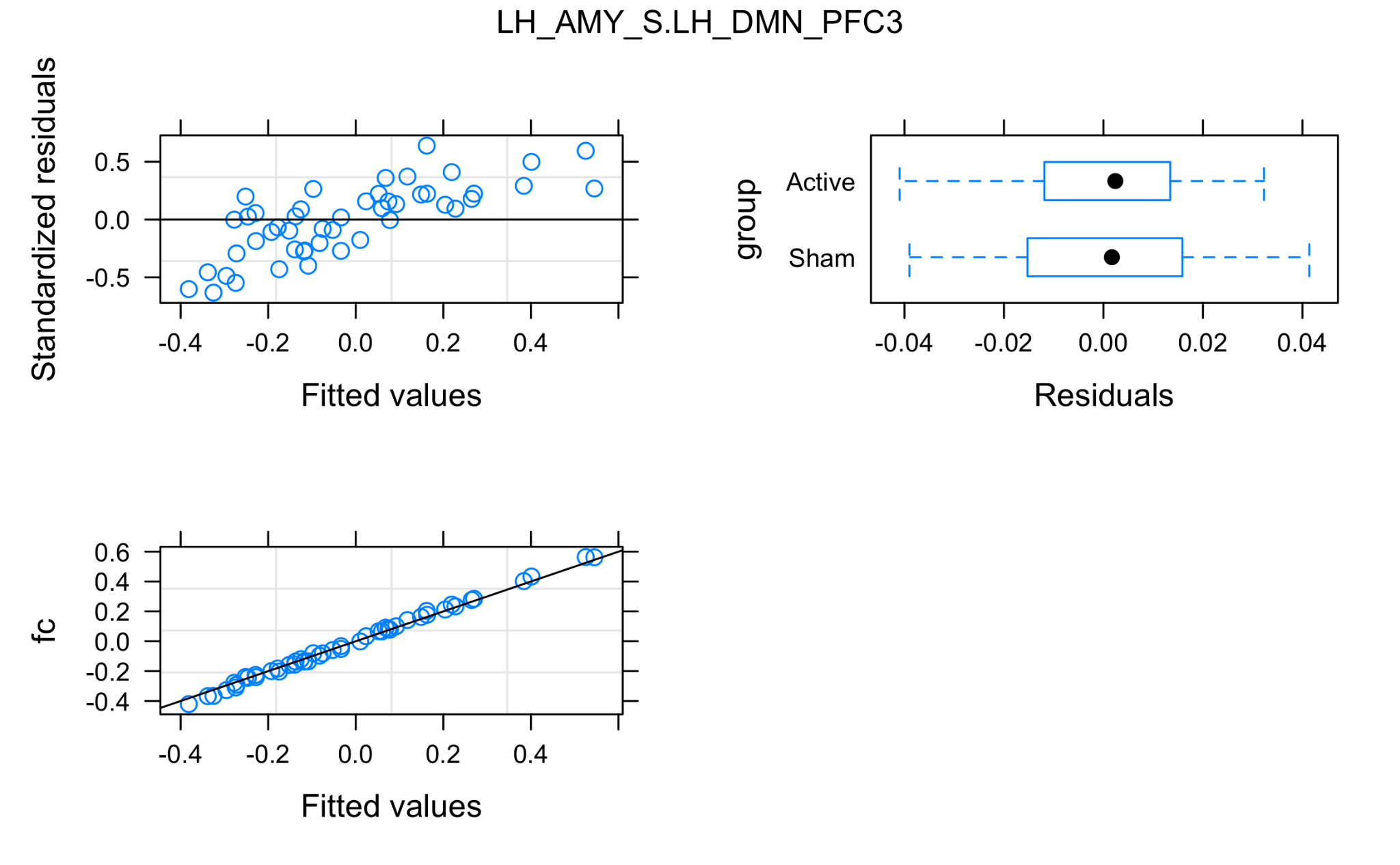


Figure s2: ANOVA model plots of significant group (active vs sham) * time (baseline vs immediate-post) interaction - *p* ≤ 0.001 from main analysis

| **hemisphere** | **network** | **roi_num** | **roiNum_network** |
| --- | --- | --- | --- |
| **LH** | VN | 1 | 1.0 |
| **LH** | VN | 2 | 2.0 |
| **LH** | VN | 3 | 3.0 |
| **LH** | VN | 4 | 4.0 |
| **LH** | VN | 5 | 5.0 |
| **LH** | VN | 6 | 6.0 |
| **LH** | VN | 7 | 7.0 |
| **LH** | VN | 8 | 8.0 |
| **LH** | VN | 9 | 9.0 |
| **LH** | SMN | 1 | 1.0 |
| **LH** | SMN | 2 | 2.0 |
| **LH** | SMN | 3 | 3.0 |
| **LH** | SMN | 4 | 4.0 |
| **LH** | SMN | 5 | 5.0 |
| **LH** | SMN | 6 | 6.0 |
| **LH** | DAN | Post1 | 1.0 |
| **LH** | DAN | Post2 | 2.0 |
| **LH** | DAN | Post3 | 3.0 |
| **LH** | DAN | Post4 | 4.0 |
| **LH** | DAN | Post5 | 5.0 |
| **LH** | DAN | Post6 | 6.0 |
| **LH** | DAN | PrCv1 | 7.0 |
| **LH** | DAN | FEF1 | 8.0 |
| **LH** | SN | ParOper1 | 1.0 |
| **LH** | SN | FrOperIns1 | 2.0 |
| **LH** | SN | FrOperIns2 | 3.0 |
| **LH** | SN | PFCl1 | 4.0 |
| **LH** | SN | Med1 | 5.0 |
| **LH** | SN | Med2 | 6.0 |
| **LH** | SN | Med3 | 7.0 |
| **LH** | LN | OFC1 | 1.0 |
| **LH** | LN | TempPole1 | 2.0 |
| **LH** | LN | TempPole2 | 3.0 |
| **LH** | CEN | Par1 | 1.0 |
| **LH** | CEN | PFCl1 | 2.0 |
| **LH** | CEN | pCun1 | 3.0 |
| **LH** | CEN | Cing1 | 4.0 |
| **LH** | DMN | Temp1 | 1.0 |
| **LH** | DMN | Temp2 | 2.0 |
| **LH** | DMN | Par1 | 3.0 |
| **LH** | DMN | Par2 | 4.0 |
| **LH** | DMN | PFC1 | 5.0 |
| **LH** | DMN | PFC2 | 6.0 |
| **LH** | DMN | PFC3 | 7.0 |
| **LH** | DMN | PFC4 | 8.0 |
| **LH** | DMN | PFC5 | 9.0 |
| **LH** | DMN | PFC6 | 10.0 |
| **LH** | DMN | PFC7 | 11.0 |
| **LH** | DMN | pCunPCC1 | 12.0 |
| **LH** | DMN | pCunPCC2 | 13.0 |
| **RH** | VN | 1 | 10.0 |
| **RH** | VN | 2 | 11.0 |
| **RH** | VN | 3 | 12.0 |
| **RH** | VN | 4 | 13.0 |
| **RH** | VN | 5 | 14.0 |
| **RH** | VN | 6 | 15.0 |
| **RH** | VN | 7 | 16.0 |
| **RH** | VN | 8 | 17.0 |
| **RH** | SMN | 1 | 7.0 |
| **RH** | SMN | 2 | 8.0 |
| **RH** | SMN | 3 | 9.0 |
| **RH** | SMN | 4 | 10.0 |
| **RH** | SMN | 5 | 11.0 |
| **RH** | SMN | 6 | 12.0 |
| **RH** | SMN | 7 | 13.0 |
| **RH** | SMN | 8 | 14.0 |
| **RH** | DAN | Post1 | 9.0 |
| **RH** | DAN | Post2 | 10.0 |
| **RH** | DAN | Post3 | 11.0 |
| **RH** | DAN | Post4 | 12.0 |
| **RH** | DAN | Post5 | 13.0 |
| **RH** | DAN | PrCv1 | 14.0 |
| **RH** | DAN | FEF1 | 15.0 |
| **RH** | SN | TempOccPar1 | 8.0 |
| **RH** | SN | TempOccPar2 | 9.0 |
| **RH** | SN | FrOperIns1 | 10.0 |
| **RH** | SN | Med1 | 11.0 |
| **RH** | SN | Med2 | 12.0 |
| **RH** | LN | OFC1 | 4.0 |
| **RH** | LN | TempPole1 | 5.0 |
| **RH** | CEN | Par1 | 5.0 |
| **RH** | CEN | Par2 | 6.0 |
| **RH** | CEN | PFCl1 | 7.0 |
| **RH** | CEN | PFCl2 | 8.0 |
| **RH** | CEN | PFCl3 | 9.0 |
| **RH** | CEN | PFCl4 | 10.0 |
| **RH** | CEN | Cing1 | 11.0 |
| **RH** | CEN | PFCmp1 | 12.0 |
| **RH** | CEN | pCun1 | 13.0 |
| **RH** | DMN | Par1 | 14.0 |
| **RH** | DMN | Temp1 | 15.0 |
| **RH** | DMN | Temp2 | 16.0 |
| **RH** | DMN | Temp3 | 17.0 |
| **RH** | DMN | PFCv1 | 18.0 |
| **RH** | DMN | PFCv2 | 19.0 |
| **RH** | DMN | PFCdPFCm1 | 20.0 |
| **RH** | DMN | PFCdPFCm2 | 21.0 |
| **RH** | DMN | PFCdPFCm3 | 22.0 |
| **RH** | DMN | pCunPCC1 | 23.0 |
| **RH** | DMN | pCunPCC2 | 24.0 |
| **LH** | dlPFC | 1 | 1.0 |
| **RH** | dlPFC | 1 | 2.0 |
| **LH** | AMY | S | 1.0 |
| **RH** | AMY | S | 2.0 |
| **LH** | AMY | CM | 3.0 |
| **RH** | AMY | CM | 4.0 |
| **LH** | AMY | LB | 5.0 |
| **RH** | AMY | LB | 6.0 |
| **LH** | STR | lim | 1.0 |
| **RH** | STR | lim | 2.0 |
| **LH** | STR | exe | 3.0 |
| **RH** | STR | exe | 4.0 |
| **LH** | STR | sensorimotor | 5.0 |
| **RH** | STR | sensorimotor | 6.0 |
| **LH** | HIP | 1 | 1.0 |
| **RH** | HIP | 1 | 2.0 |
| **LH** | THAL | 1 | 1.0 |
| **RH** | THAL | 1 | 2.0 |

Table s9: List of ROIs that have been included in the imaging analysis based on the Schaeffer parcellation atlas.

| **FC Pairs name** | **r** | **p** |
| --- | --- | --- |
| **LH_VN_4.LH_VN_3** | 0.408 | 0.048 |
| **LH_VN_7.LH_VN_5** | 0.572 | 0.003 |
| **LH_VN_8.LH_VN_3** | 0.477 | 0.019 |
| **LH_VN_8.LH_VN_5** | 0.537 | 0.007 |
| **LH_DAN_Post1.LH_VN_8** | -0.459 | 0.024 |
| **LH_DAN_Post1.LH_VN_9** | -0.41 | 0.047 |
| **LH_DAN_Post1.LH_SMN_2** | -0.428 | 0.037 |
| **LH_DAN_Post2.LH_VN_8** | -0.473 | 0.02 |
| **LH_DAN_Post3.LH_SMN_6** | -0.441 | 0.031 |
| **LH_DAN_Post4.LH_VN_8** | -0.429 | 0.037 |
| **LH_DAN_Post5.LH_VN_2** | 0.405 | 0.05 |
| **LH_DAN_Post6.LH_VN_7** | 0.413 | 0.045 |
| **LH_DAN_PrCv1.LH_SMN_3** | 0.41 | 0.047 |
| **LH_DAN_PrCv1.LH_DAN_Post6** | -0.427 | 0.038 |
| **LH_SN_ParOper1.LH_VN_8** | -0.554 | 0.005 |
| **LH_SN_FrOperIns1.LH_VN_5** | 0.419 | 0.041 |
| **LH_SN_Med2.LH_SN_ParOper1** | -0.431 | 0.035 |
| **LH_SN_Med2.LH_SN_FrOperIns1** | -0.47 | 0.021 |
| **LH_SN_Med3.LH_VN_6** | 0.408 | 0.048 |
| **LH_SN_Med3.LH_VN_7** | 0.495 | 0.014 |
| **LH_SN_Med3.LH_DAN_FEF1** | 0.455 | 0.025 |
| **LH_LN_OFC1.LH_VN_8** | 0.495 | 0.014 |
| **LH_LN_TempPole1.LH_SMN_4** | 0.441 | 0.031 |
| **LH_CEN_Par1.LH_DAN_Post6** | -0.406 | 0.049 |
| **LH_CEN_PFCl1.LH_SMN_3** | 0.422 | 0.04 |
| **LH_CEN_PFCl1.LH_SMN_4** | 0.409 | 0.047 |
| **LH_CEN_PFCl1.LH_DAN_Post6** | -0.473 | 0.019 |
| **LH_CEN_PFCl1.LH_LN_OFC1** | 0.474 | 0.019 |
| **LH_CEN_Cing1.LH_DAN_PrCv1** | -0.415 | 0.044 |
| **LH_DMN_Temp1.LH_SMN_4** | 0.408 | 0.048 |
| **LH_DMN_Par1.LH_VN_5** | -0.429 | 0.037 |
| **LH_DMN_Par2.LH_VN_1** | 0.452 | 0.026 |
| **LH_DMN_PFC2.LH_LN_OFC1** | 0.502 | 0.012 |
| **LH_DMN_PFC3.LH_VN_8** | 0.431 | 0.036 |
| **LH_DMN_PFC4.LH_DAN_PrCv1** | 0.41 | 0.046 |
| **LH_DMN_PFC5.LH_DAN_FEF1** | 0.557 | 0.005 |
| **LH_DMN_PFC5.LH_CEN_PFCl1** | 0.503 | 0.012 |
| **LH_DMN_PFC6.LH_LN_TempPole1** | -0.482 | 0.017 |
| **LH_DMN_PFC7.LH_LN_TempPole1** | -0.43 | 0.036 |
| **RH_VN_1.LH_DAN_Post1** | 0.421 | 0.041 |
| **RH_VN_2.LH_VN_5** | 0.525 | 0.008 |
| **RH_VN_3.LH_DMN_PFC4** | 0.491 | 0.015 |
| **RH_VN_4.LH_VN_3** | 0.481 | 0.017 |
| **RH_VN_4.LH_VN_8** | 0.492 | 0.015 |
| **RH_VN_4.LH_VN_9** | 0.528 | 0.008 |
| **RH_VN_4.LH_DMN_PFC1** | -0.421 | 0.041 |
| **RH_VN_4.RH_VN_3** | 0.47 | 0.021 |
| **RH_VN_5.LH_VN_5** | 0.436 | 0.033 |
| **RH_VN_6.LH_DAN_Post6** | 0.503 | 0.012 |
| **RH_VN_7.LH_VN_3** | 0.407 | 0.048 |
| **RH_VN_7.LH_VN_5** | 0.489 | 0.015 |
| **RH_VN_7.RH_VN_2** | -0.445 | 0.029 |
| **RH_VN_7.RH_VN_5** | 0.512 | 0.011 |
| **RH_VN_8.LH_SMN_4** | -0.508 | 0.011 |
| **RH_SMN_1.LH_DMN_PFC5** | -0.405 | 0.05 |
| **RH_SMN_2.LH_LN_OFC1** | -0.421 | 0.041 |
| **RH_SMN_2.LH_CEN_Cing1** | -0.464 | 0.022 |
| **RH_SMN_3.LH_LN_OFC1** | -0.419 | 0.042 |
| **RH_SMN_6.RH_VN_3** | -0.462 | 0.023 |
| **RH_SMN_7.LH_VN_4** | -0.536 | 0.007 |
| **RH_SMN_7.LH_DMN_PFC5** | 0.541 | 0.006 |
| **RH_SMN_8.LH_DAN_Post6** | 0.497 | 0.014 |
| **RH_SMN_8.LH_DMN_PFC1** | 0.422 | 0.04 |
| **RH_DAN_Post1.LH_VN_8** | -0.535 | 0.007 |
| **RH_DAN_Post1.LH_VN_9** | -0.502 | 0.012 |
| **RH_DAN_Post1.LH_DAN_FEF1** | -0.433 | 0.034 |
| **RH_DAN_Post1.RH_VN_4** | -0.454 | 0.026 |
| **RH_DAN_Post1.RH_VN_5** | -0.428 | 0.037 |
| **RH_DAN_Post3.LH_VN_8** | -0.481 | 0.017 |
| **RH_DAN_Post4.LH_VN_8** | -0.436 | 0.033 |
| **RH_DAN_Post4.LH_DMN_PFC3** | 0.405 | 0.05 |
| **RH_DAN_Post4.RH_VN_8** | -0.503 | 0.012 |
| **RH_DAN_Post5.LH_SN_FrOperIns1** | -0.407 | 0.048 |
| **RH_DAN_Post5.LH_LN_TempPole1** | 0.444 | 0.03 |
| **RH_SN_TempOccPar1.LH_VN_9** | -0.429 | 0.036 |
| **RH_SN_TempOccPar1.LH_SMN_1** | -0.485 | 0.016 |
| **RH_SN_TempOccPar1.LH_DAN_Post4** | -0.433 | 0.034 |
| **RH_SN_TempOccPar1.RH_SMN_1** | -0.449 | 0.028 |
| **RH_SN_FrOperIns1.LH_SMN_6** | 0.414 | 0.044 |
| **RH_SN_Med1.LH_DAN_PrCv1** | -0.444 | 0.03 |
| **RH_SN_Med1.LH_SN_FrOperIns1** | -0.539 | 0.007 |
| **RH_SN_Med1.LH_SN_PFCl1** | -0.433 | 0.035 |
| **RH_SN_Med2.LH_SN_ParOper1** | -0.459 | 0.024 |
| **RH_SN_Med2.RH_SMN_8** | -0.488 | 0.016 |
| **RH_LN_OFC1.LH_DMN_PFC7** | -0.695 | 0 |
| **RH_LN_OFC1.RH_SN_TempOccPar2** | 0.589 | 0.002 |
| **RH_LN_TempPole1.LH_SMN_2** | 0.433 | 0.034 |
| **RH_LN_TempPole1.LH_SMN_4** | 0.526 | 0.008 |
| **RH_LN_TempPole1.RH_SMN_4** | 0.446 | 0.029 |
| **RH_CEN_Par1.LH_VN_8** | -0.455 | 0.026 |
| **RH_CEN_Par1.LH_DAN_Post6** | -0.44 | 0.031 |
| **RH_CEN_Par2.LH_DMN_PFC7** | -0.417 | 0.043 |
| **RH_CEN_Par2.RH_VN_8** | -0.432 | 0.035 |
| **RH_CEN_PFCl1.LH_SMN_5** | -0.455 | 0.025 |
| **RH_CEN_PFCl3.RH_LN_TempPole1** | 0.407 | 0.049 |
| **RH_CEN_PFCl4.RH_LN_OFC1** | -0.416 | 0.043 |
| **RH_CEN_PFCmp1.LH_DMN_PFC6** | -0.427 | 0.037 |
| **RH_CEN_PFCmp1.RH_DAN_Post5** | 0.474 | 0.019 |
| **RH_CEN_PFCmp1.RH_SN_TempOccPar1** | 0.418 | 0.042 |
| **RH_CEN_pCun1.LH_SMN_5** | -0.427 | 0.038 |
| **RH_CEN_pCun1.LH_DMN_PFC7** | -0.432 | 0.035 |
| **RH_DMN_Par1.LH_DMN_PFC3** | -0.441 | 0.031 |
| **RH_DMN_Par1.LH_DMN_PFC6** | -0.443 | 0.03 |
| **RH_DMN_Temp1.LH_DMN_pCunPCC2** | -0.437 | 0.033 |
| **RH_DMN_Temp1.RH_VN_1** | -0.502 | 0.013 |
| **RH_DMN_Temp2.RH_SN_TempOccPar2** | 0.411 | 0.046 |
| **RH_DMN_Temp3.LH_VN_2** | 0.524 | 0.009 |
| **RH_DMN_Temp3.LH_VN_5** | -0.478 | 0.018 |
| **RH_DMN_Temp3.LH_DMN_Par2** | 0.544 | 0.006 |
| **RH_DMN_PFCv1.LH_LN_TempPole1** | -0.417 | 0.043 |
| **RH_DMN_PFCv1.LH_DMN_PFC3** | -0.51 | 0.011 |
| **RH_DMN_PFCv1.LH_DMN_PFC6** | -0.449 | 0.028 |
| **RH_DMN_PFCv1.LH_DMN_PFC7** | -0.404 | 0.05 |
| **RH_DMN_PFCv1.RH_VN_4** | 0.55 | 0.005 |
| **RH_DMN_PFCv1.RH_DAN_Post4** | 0.464 | 0.023 |
| **RH_DMN_PFCv1.RH_DAN_Post5** | 0.66 | 0 |
| **RH_DMN_PFCv1.RH_DAN_PrCv1** | 0.525 | 0.008 |
| **RH_DMN_PFCv1.RH_SN_TempOccPar1** | 0.422 | 0.04 |
| **RH_DMN_PFCv1.RH_CEN_PFCl3** | 0.59 | 0.002 |
| **RH_DMN_PFCv2.LH_DMN_PFC5** | -0.444 | 0.03 |
| **RH_DMN_PFCv2.RH_DAN_Post5** | 0.514 | 0.01 |
| **RH_DMN_PFCdPFCm1.LH_VN_9** | 0.454 | 0.026 |
| **RH_DMN_PFCdPFCm1.LH_SN_FrOperIns2** | 0.424 | 0.039 |
| **RH_DMN_PFCdPFCm1.LH_CEN_pCun1** | -0.408 | 0.048 |
| **RH_DMN_PFCdPFCm1.LH_DMN_PFC4** | -0.63 | 0.001 |
| **RH_DMN_PFCdPFCm1.LH_DMN_PFC5** | -0.467 | 0.022 |
| **RH_DMN_PFCdPFCm1.LH_DMN_PFC6** | -0.429 | 0.036 |
| **RH_DMN_PFCdPFCm1.LH_DMN_PFC7** | -0.497 | 0.014 |
| **RH_DMN_PFCdPFCm1.LH_DMN_pCunPCC1** | -0.515 | 0.01 |
| **RH_DMN_PFCdPFCm1.RH_SN_TempOccPar2** | 0.44 | 0.031 |
| **RH_DMN_PFCdPFCm2.LH_DMN_PFC6** | -0.519 | 0.009 |
| **RH_DMN_PFCdPFCm3.LH_VN_5** | -0.455 | 0.026 |
| **RH_DMN_PFCdPFCm3.LH_DMN_PFC5** | -0.428 | 0.037 |
| **RH_DMN_PFCdPFCm3.RH_CEN_PFCmp1** | -0.47 | 0.02 |
| **RH_DMN_pCunPCC1.LH_VN_2** | 0.413 | 0.045 |
| **RH_DMN_pCunPCC1.RH_VN_4** | -0.43 | 0.036 |
| **RH_DMN_pCunPCC1.RH_VN_5** | -0.504 | 0.012 |
| **RH_DMN_pCunPCC1.RH_VN_8** | -0.472 | 0.02 |
| **RH_DMN_pCunPCC1.RH_SMN_7** | 0.453 | 0.026 |
| **RH_DMN_pCunPCC1.RH_DAN_Post1** | 0.418 | 0.042 |
| **RH_DMN_pCunPCC2.LH_VN_5** | -0.445 | 0.029 |
| **RH_DMN_pCunPCC2.RH_SN_Med2** | 0.412 | 0.046 |
| **LH_dlPFC_1.LH_DAN_Post2** | 0.479 | 0.018 |
| **LH_dlPFC_1.LH_DAN_FEF1** | 0.515 | 0.01 |
| **LH_dlPFC_1.LH_LN_OFC1** | 0.497 | 0.013 |
| **LH_dlPFC_1.LH_LN_TempPole1** | -0.413 | 0.045 |
| **LH_dlPFC_1.LH_CEN_PFCl1** | 0.489 | 0.015 |
| **LH_dlPFC_1.LH_CEN_pCun1** | 0.451 | 0.027 |
| **LH_dlPFC_1.RH_LN_OFC1** | 0.413 | 0.045 |
| **LH_dlPFC_1.RH_CEN_pCun1** | -0.413 | 0.045 |
| **LH_dlPFC_1.RH_DMN_Temp1** | -0.62 | 0.001 |
| **LH_dlPFC_1.RH_DMN_PFCv2** | -0.499 | 0.013 |
| **LH_dlPFC_1.RH_DMN_PFCdPFCm1** | -0.413 | 0.045 |
| **LH_dlPFC_1.RH_DMN_PFCdPFCm2** | -0.633 | 0.001 |
| **LH_dlPFC_1.RH_DMN_PFCdPFCm3** | -0.609 | 0.002 |
| **RH_dlPFC_1.LH_VN_1** | -0.409 | 0.047 |
| **RH_dlPFC_1.LH_DMN_PFC5** | -0.421 | 0.04 |
| **RH_dlPFC_1.LH_DMN_PFC6** | -0.486 | 0.016 |
| **RH_dlPFC_1.RH_DAN_FEF1** | 0.463 | 0.023 |
| **RH_dlPFC_1.RH_CEN_Cing1** | 0.483 | 0.017 |
| **LH_AMY_S.LH_VN_1** | -0.507 | 0.011 |
| **LH_AMY_S.LH_DMN_Par2** | -0.423 | 0.04 |
| **LH_AMY_S.RH_dlPFC_1** | -0.418 | 0.042 |
| **RH_AMY_S.LH_VN_7** | -0.464 | 0.022 |
| **RH_AMY_S.LH_SMN_1** | -0.423 | 0.04 |
| **RH_AMY_S.RH_VN_6** | -0.571 | 0.004 |
| **RH_AMY_S.RH_DMN_PFCv1** | 0.472 | 0.02 |
| **LH_AMY_CM.LH_DAN_PrCv1** | 0.438 | 0.032 |
| **LH_AMY_CM.LH_SN_Med3** | 0.414 | 0.044 |
| **LH_AMY_CM.LH_CEN_Par1** | -0.405 | 0.049 |
| **LH_AMY_CM.RH_DAN_Post5** | 0.407 | 0.048 |
| **RH_AMY_CM.LH_SN_Med3** | 0.502 | 0.012 |
| **RH_AMY_CM.LH_DMN_pCunPCC1** | -0.573 | 0.003 |
| **RH_AMY_CM.RH_DAN_Post4** | 0.477 | 0.018 |
| **RH_AMY_CM.RH_SN_Med2** | 0.61 | 0.002 |
| **RH_AMY_CM.RH_CEN_Par2** | 0.501 | 0.013 |
| **RH_AMY_CM.RH_CEN_PFCl4** | 0.634 | 0.001 |
| **RH_AMY_CM.LH_AMY_S** | 0.534 | 0.007 |
| **LH_AMY_LB.LH_DAN_Post1** | 0.45 | 0.027 |
| **LH_AMY_LB.LH_SN_Med3** | 0.46 | 0.024 |
| **LH_AMY_LB.RH_SN_FrOperIns1** | 0.406 | 0.049 |
| **LH_AMY_LB.RH_SN_Med1** | 0.484 | 0.017 |
| **LH_AMY_LB.RH_CEN_Par1** | -0.472 | 0.02 |
| **RH_AMY_LB.LH_VN_5** | -0.611 | 0.002 |
| **RH_AMY_LB.LH_DAN_PrCv1** | 0.44 | 0.032 |
| **RH_AMY_LB.LH_SN_Med3** | 0.503 | 0.012 |
| **RH_AMY_LB.LH_DMN_Temp1** | 0.491 | 0.015 |
| **RH_AMY_LB.LH_DMN_PFC4** | 0.412 | 0.046 |
| **RH_AMY_LB.RH_SMN_2** | 0.405 | 0.049 |
| **RH_AMY_LB.RH_SMN_8** | 0.491 | 0.015 |
| **RH_AMY_LB.RH_SN_Med2** | 0.408 | 0.048 |
| **RH_AMY_LB.RH_DMN_PFCv1** | 0.441 | 0.031 |
| **LH_STR_lim.LH_SMN_6** | 0.428 | 0.037 |
| **LH_STR_lim.LH_CEN_PFCl1** | -0.431 | 0.035 |
| **RH_STR_lim.LH_DAN_PrCv1** | 0.471 | 0.02 |
| **RH_STR_lim.LH_CEN_Par1** | -0.461 | 0.023 |
| **RH_STR_lim.RH_CEN_PFCl1** | -0.462 | 0.023 |
| **RH_STR_lim.RH_CEN_PFCmp1** | -0.524 | 0.009 |
| **LH_STR_exe.RH_CEN_PFCl1** | -0.475 | 0.019 |
| **LH_STR_exe.RH_CEN_Cing1** | -0.451 | 0.027 |
| **RH_STR_.LH_CEN_Cing1** | -0.437 | 0.033 |
| **RH_STR_.RH_CEN_Cing1** | -0.407 | 0.048 |
| **RH_STR_.RH_CEN_PFCmp1** | -0.482 | 0.017 |
| **RH_STR_.LH_STR_lim** | 0.433 | 0.035 |
| **LH_STR_sensorimotor.LH_SN_PFCl1** | 0.544 | 0.006 |
| **LH_STR_sensorimotor.RH_SMN_7** | -0.407 | 0.049 |
| **LH_HIP_1.LH_VN_1** | 0.472 | 0.02 |
| **LH_HIP_1.RH_SMN_2** | 0.415 | 0.044 |
| **LH_HIP_1.RH_SMN_3** | 0.405 | 0.05 |
| **LH_HIP_1.RH_SMN_4** | 0.448 | 0.028 |
| **LH_HIP_1.RH_DMN_PFCv1** | 0.429 | 0.036 |
| **RH_HIP_1.LH_DMN_pCunPCC1** | -0.583 | 0.003 |
| **RH_HIP_1.RH_VN_4** | 0.413 | 0.045 |
| **RH_HIP_1.RH_SMN_2** | 0.458 | 0.024 |
| **RH_HIP_1.RH_SN_TempOccPar2** | 0.422 | 0.04 |
| **RH_HIP_1.RH_DMN_PFCv1** | 0.468 | 0.021 |
| **RH_HIP_1.RH_dlPFC_1** | -0.429 | 0.037 |
| **LH_THAL_1.LH_DMN_PFC7** | -0.42 | 0.041 |
| **LH_THAL_1.LH_STR_lim** | 0.444 | 0.03 |
| **RH_THAL_1.LH_SN_ParOper1** | -0.492 | 0.015 |
| **RH_THAL_1.LH_CEN_PFCl1** | -0.602 | 0.002 |
| **RH_THAL_1.RH_AMY_S** | -0.53 | 0.008 |
| **RH_THAL_1.LH_THAL_1** | -0.435 | 0.034 |

Table s10: Functional Connectivity (FC) pairs that elicited non-specific (no active/sham group effect) significant relationship between FC change and MADRS change at immediate-post visit. r=Pearson’s coefficient, p=p-value.

|  | MNI coordinates | | |  |
| --- | --- | --- | --- | --- |
| subject ID | x | y | z | Schaefer network name |
| sub-B001 | -40 | 42 | 12 | LH_Cont_PFCl_1 |
| sub-B003 | -44 | 32 | 16 | LH_Cont_PFCl_1 |
| sub-B005 | -46 | 36 | 26 | LH_Cont_PFCl_1 |
| sub-B007 | -52 | 34 | 16 | LH_Cont_PFCl_1 |
| sub-B008 | -44 | 50 | 14 | LH_Cont_PFCl_1 |
| sub-B009 | -44 | 44 | 14 | LH_Cont_PFCl_1 |
| sub-B011 | -50 | 42 | 14 | LH_Cont_PFCl_1 |
| sub-B013 | -52 | 26 | 24 | LH_Cont_PFCl_1 |
| sub-B014 | -44 | 46 | 14 | LH_Cont_PFCl_1 |
| sub-B015 | -46 | 42 | 14 | LH_Cont_PFCl_1 |
| sub-B016 | -44 | 44 | 12 | LH_Cont_PFCl_1 |
| sub-B017 | -48 | 38 | 14 | LH_Cont_PFCl_1 |
| sub-B018 | -52 | 24 | 24 | LH_Cont_PFCl_1 |
| sub-B020 | -52 | 34 | 18 | LH_Cont_PFCl_1 |
| sub-B021 | -48 | 28 | 16 | LH_Cont_PFCl_1 |
| sub-B022 | -50 | 34 | 16 | LH_Cont_PFCl_1 |
| sub-B023 | -42 | 42 | 28 | LH_Cont_PFCl_1 |
| sub-B024 | -46 | 46 | 8 | LH_Cont_PFCl_1 |
| sub-B025 | -44 | 34 | 16 | LH_Cont_PFCl_1 |
| sub-B026 | -52 | 32 | 24 | LH_Cont_PFCl_1 |
| sub-B027 | -46 | 28 | 24 | LH_Cont_PFCl_1 |
| sub-B031 | -44 | 38 | 16 | LH_Cont_PFCl_1 |
| sub-B032 | -44 | 32 | 26 | LH_Cont_PFCl_1 |

Table s11: MNI coordinates of targets and corresponding Schaefer’s parcellation network ROI those landed.


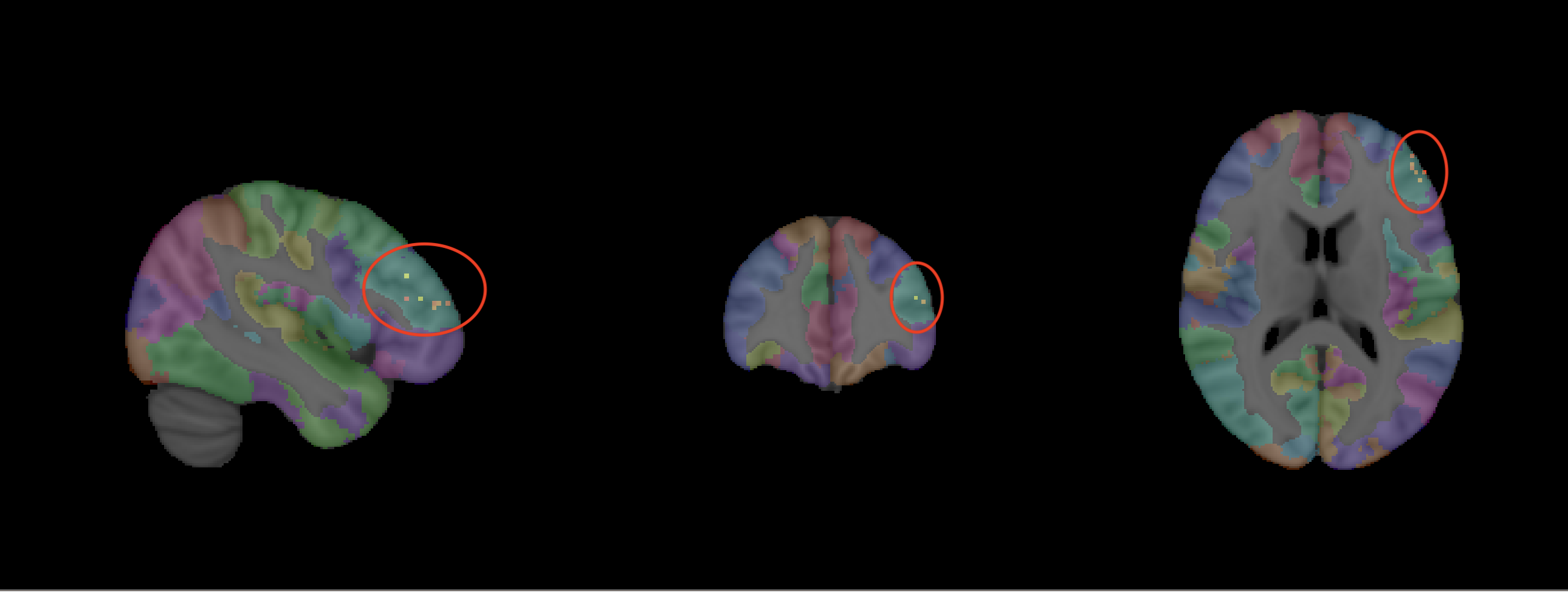


Figure s3: illustration of individual targets (dots within red circle) mapped onto Schaefer’s brain parcellation. Note that all targets landed within the LH_Cont_PFCl_1 network corresponding to the lateral prefrontal cortex of the left hemisphere (control executive network)
